# Engineering a 3D in vitro model of human skeletal muscle at the single fiber scale

**DOI:** 10.1371/journal.pone.0232081

**Published:** 2020-05-06

**Authors:** Anna Urciuolo, Elena Serena, Rusha Ghua, Susi Zatti, Monica Giomo, Nicolò Mattei, Massimo Vetralla, Giulia Selmin, Camilla Luni, Nicola Vitulo, Giorgio Valle, Libero Vitiello, Nicola Elvassore

**Affiliations:** 1 Industrial Engineering Department, University of Padova, Padova, Italy; 2 Venetian Institute of Molecular Medicine, Padova, Italy; 3 Women’s and Children’s Health Department, University of Padova, Padova, Italy; 4 Department of Biology, University of Padova, Padova, Italy; 5 Shanghai Institute for Advanced Immunochemical Studies, ShanghaiTech University, Shanghai, China; 6 Department of Biotechnologies, University of Verona, Verona, Italy; 7 Interuniversity Institute of Myology (IIM), Assisi, Italy; 8 University College London ICH, London, England, United Kingdom; University of Minnesota Medical School, UNITED STATES

## Abstract

The reproduction of reliable *in vitro* models of human skeletal muscle is made harder by the intrinsic 3D structural complexity of this tissue. Here we coupled engineered hydrogel with 3D structural cues and specific mechanical properties to derive human 3D muscle constructs (“myobundles”) at the scale of single fibers, by using primary myoblasts or myoblasts derived from embryonic stem cells. To this aim, cell culture was performed in confined, laminin-coated micrometric channels obtained inside a 3D hydrogel characterized by the optimal stiffness for skeletal muscle myogenesis. Primary myoblasts cultured in our 3D culture system were able to undergo myotube differentiation and maturation, as demonstrated by the proper expression and localization of key components of the sarcomere and sarcolemma. Such approach allowed the generation of human myobundles of ~10 mm in length and ~120 μm in diameter, showing spontaneous contraction 7 days after cell seeding. Transcriptome analyses showed higher similarity between 3D myobundles and skeletal signature, compared to that found between 2D myotubes and skeletal muscle, mainly resulting from expression in 3D myobundles of categories of genes involved in skeletal muscle maturation, including extracellular matrix organization. Moreover, imaging analyses confirmed that structured 3D culture system was conducive to differentiation/maturation also when using myoblasts derived from embryonic stem cells. In conclusion, our structured 3D model is a promising tool for modelling human skeletal muscle in healthy and diseases conditions.

## 1. Introduction

Defined as the most abundant tissue in the human body [1], skeletal muscle is a dynamic and complex tissue in which the three-dimensional (3D) organization of myofibers is guaranteed by the presence of a complex tubular network of extracellular matrix [2,3]. Despite possessing good regenerative properties, skeletal muscle can be functionally compromised due to neuromuscular or metabolic diseases, as well as traumatic injuries [4]. The possibility to develop 3D *in vitro* models of human skeletal muscle would provide an important tool for basic biological studies and development of regenerative medicine strategies, as well as of drug screening for muscle disorders [5,6]. The need for muscle 3D models resides in the intrinsic structural complexity of this tissue, which in turn influences its function. Highly packed and aligned myotubes with a proper 3D organization as well as the presence of a mature contractile system represent the main basic requirement for the development of a valid *in vitro* model. Therefore, in recent years tissue engineering strategies have been developed to generate 3D skeletal muscle *in vitro* cultures by using natural and synthetic materials, including fibrin [7–12], alginate [13,14], polycaprolactone (PCL)-based polymers [15–17], and decellularized matrix [18]. Anisotropic environments promote *in vitro* alignment of myogenic cells and favor their fusion, myogenesis and maturation. Nanofibers, anchors and hydrogel compaction, chemical surface patterning, stencils, mechanical stimulations, and electrical or magnetic fields are some of the main strategies used to allow the generation of anisotropic environments for skeletal muscle in vitro culture [19]. Moreover, scaffolds with specific stiffness, electrical conductivity, polymeric compositions, and soluble factors have been developed to improve skeletal muscle cell differentiation [19]. The majority of the studies for *in vitro* engineering skeletal muscle have been made by using rodent skeletal muscular cells [9,20–26]. Fewer groups reported the use of primary myogenic human cells, either alone or combined with other cell types [12,27–33], and only recently a couple of studies introduced human pluripotent stem cells (hPSCs) for 3D modelling skeletal muscle [34,35]. Generally, 3D muscle cultures allow longer culture times together with increased maturation of myotubes in terms of size, protein content and improved maturation of myosin heavy chain (MHC) gene expression compared to 2D cultures [36].

In the present study, we coupled topological and mechanical properties of a synthetic scaffolds to derive and characterize 3D culture of single human myobundles starting from primary human myogenic cells. More in detail, cells were cultured in confined channels obtained inside a 3D hydrogel whose stiffness was optimized for skeletal muscle myogenesis. This 3D culture system allowed the generation of human myobundles of ~10 mm in length and with a diameter comparable to that of single fibers. These constructs were able to spontaneously contract and showed markers of myotube maturation during the culture period, as also confirmed by transcriptome analysis. Importantly, we also demonstrated that in our 3D system human myobundles could also be obtained by using myoblasts derived from human embryonic stem cells (hESC-derived myoblasts).

## 2. Materials and methods

Please notice that no specific ethic approval was needed for the present study, as the human cells used were provided by Institutions that had already acquired the appropriate consents at the time of initial preparation.

### 2.1 Mold preparation

A polydimethylsiloxane (PDMS) base and a curing agent (Sylgard 184, Dow Corning) were mixed together with a 16:1 ratio. The solution was placed under vacuum for 10 minutes and poured into a petri culture dish to obtain a 3mm-thick layer, then placed in an oven at 100 °C for 1 hour. After cooling, small frames of 20 x 10 x 3 mm were cut out from the slab.

### 2.2 3D Hydrogel preparation

Glass coverslips (25mm diameter, Menzel Gläser) were chemically modified to ensure the covalent binding of 3D hydrogel to the surface. Coverslips were washed with ethanol, dried at room temperature and treated with plasma for 2 minutes at 3 x 10^−1^ mbar. They were then covered with an alkoxysilane solution (95% ethanol, 3% acetic acid, 0.3% 3-(trimethoxysilyl)propyl methacrylate, Sigma Aldrich) for 3 minutes, washed with distilled water and sterilized with 70% ethanol at room temperature. Hydrogels were prepared using a phosphate-buffer-saline (PBS, Sigma Aldrich) solution containing 10% of 29:1 acrylamide-bis-acrylamide (Sigma Aldrich), 1% ammonium persulfate (APS, Sigma Aldrich) and 0.1% N,N,N′,N′-Tetramethylethylenediamine (TEMED, Sigma Aldrich). The ratio of the different components had been previously determined experimentally, to obtain an hydrogel with an elastic modulus of 15 kPa [37]. Freshly made solution was then rapidly poured into a mold prepared by stacking two PDMS frames on top of a round coverslip prepared as described above. In order to obtain the channels, tensed nylon threads of the desired diameter were placed between the two autoclaved PDMS frames. A non-treated glass coverslip was then placed on top of the mold, to exclude the presence of air and allow homogeneous polymerization, which took place in about 10 minutes. Upon gelification, threads were cut on one side and carefully pulled out from the other end, before disassembling the stacked frames.

### 2.3 3D hydrogel sterilization and laminin absorption

3D hydrogels bonded to glass slides were immersed in PBS solution (Sigma Aldrich) for 48 hours to ensure the complete removal of unreacted reagents, to avoid cytotoxicity, then sterilized under UV-light in a cell culture hood for 30 minutes making sure to reduce the liquid covering to a film. After sterilization, PBS was removed, and the parallelepiped hydrogel was left under the hood for about 15 minutes to achieve partial de-hydration. The internal surface of the micro-channels was then coated by injecting in each of them a 100 μg/mL laminin aqueous solution (Sigma Aldrich, L2020) and letting it become absorbed into the hydrogel (about 15 minutes); such procedure was repeated twice per each channel and then the whole hydrogel was rinsed with cell culture medium.

### 2.4 Cell isolation and culture

Unless otherwise indicated, all media were from Gibco-Invitrogen. The murine skeletal muscle immortalized cell line C2C12 (ATCC) was expanded at 37 °C 5% CO_2_, using Dulbecco’s modified Eagle’s medium (DMEM, Sigma-Aldrich) supplemented with 10% fetal bovine serum (FBS) and 1% penicillin−streptomycin mix solution. Differentiation was induced by differentiation medium (DMEM supplemented with 2% horse serum (HS) and 1% penicillin−streptomycin).

Human primary myoblasts were provided by the ‘‘Telethon BioBank” (Telethon Research Service, Istituto Nazionale Neurologico ‘‘Carlo Besta”, Milano, Italy). Myoblasts were expanded at 37 °C 5% CO_2_ in proliferation medium prepared with 60% High-Glucose DMEM, 20% M199 Medium (Sigma-Aldrich), 20% FBS, 10 ng/mL EGF (human recombinant epithelial growth factor, PeproTech), 2 ng/mL β-FGF (human recombinant basic fibroblast growth factor, PeproTech), 10 μg/mL insulin (from bovine pancreas, Sigma-Aldrich) and 1% penicillin-streptomycin−glutamine mix solution. Cells were cultured in standard 100 mm tissue culture Petri dishes coated with 0.5% gelatin solution (from porcine skin, Sigma-Aldrich). Differentiation was induced in confluent cultures using differentiation medium composed of DMEM Glutamax, 2% HS, 30 μg/mL insulin and 1% penicillin−streptomycin−glutamine mix.

Human embryonic stem cell-derived myoblasts (hESC-Mbs) were provided by Genea Biocells Laboratory (San Diego, CA, USA) and cultured in their proprietary Skeletal Myoblasts Medium. Differentiation was promoted by culture in Myotube Medium supplemented with 10% Myotube Maintenance Cocktail (both from Genea Biocells).

### 2.5 3D cell culture

Cells were detached from their expansion plates with trypsin-EDTA 0.25%, pelleted by centrifugation at 400g and resuspended at high density (2 x 10^5^ cells/μl) in matrigel^®^ (BD Bioscience):DMEM 1:1 solution in ice. They were then collected with a 100 μL syringe (Hamilton) and injected in each micro-channel with the aid of a stereomicroscope. Seeded hydrogels were completely immersed into proliferative medium and incubated at 37 °C and 5% of CO_2_. After 1 day, proliferation medium was replaced with differentiation medium until samples were analysed as reported in the corresponding figure legend.

### 2.6 Immunofluorescence analysis

3D hydrogels with or without cells were fixed with 4% paraformaldehyde (PFA) in PBS for 40 minutes at room temperature, rinsed in PBS, saturated in 30% sucrose in PBS 1X overnight, embedded in OCT matrix and snap-frozen in liquid nitrogen. Cross-sections of 20 μm thickness were prepared using a cryotome and used directly for immunofluorescence staining or stored at -80°C. Cryo-sections of cell-containing hydrogels were permeabilized with 0.5% Triton X-100 in PBS for 15 minutes and blocked in 10% HS for 45 minutes. Sections were incubated with primary antibodies diluted in 10% HS in PBS. For longitudinal myobundle staining, myobundles were removed from non-fixed hydrogels by dissection under a stereomicroscope, glued over a Super Frost Glass slide (Menzel gläser) and fixed with a 2% PFA for 10 minutes at room temperature. Samples were then treated with 0.5% Triton X-100 in PBS for 15 minutes, washed in PBS, blocked in 10% HS 0.1% Triton X-100 in PBS for 45 minutes, washed in 0.1% Triton X-100 in PBS and finally incubated with primary antibodies diluted in 10% HS 0.1% Triton X-100 in PBS.

Primary antibodies were incubated either at 4°C overnight or 37 °C for 1 hour according to manufacturers’ instructions. The following primary antibodies, all from Santa Cruz Biotechnology, diluted 1:100 in PBS 3% BSA, were used: α-actinin (mouse monoclonal, Sigma A7811), desmin (rabbit polyclonal, Sigma D8281); dystrophin (rabbit polyclonal, Abcam ab15277); laminin (rabbit polyclonal, Sigma, L9393); vinculin (mouse monoclonal, Santa Cruz sc73614); adult MHC (mouse monoclonal, Sigma M1570). Secondary antibodies, anti-mouse Alexa Fluor 594 and anti-rabbit Alexa Fluor 488 (both from Santa Cruz Biotechnology, 1:200 dilution), were incubated for 45 min at 37°C. Nuclei were counterstained with DAPI, samples were mounted with Fluoroshield^™^ (Sigma Aldrich) and analyzed using an epifluorescence microscope (DMI6000B Leica, for cross sections) or a confocal microscope (SP5 Leica; for isolated myobundles).

### 2.7 RNA extraction, sequencing and analysis

Analyses were performed on myoblasts (n = 2), 2D myotubes (n = 2), 3D myobundles (n = 1; 12 myobundles pooled together) and from two additional mRNA samples of adult human skeletal muscle from commercial suppliers (AMSBIO and Stratagene).

RNA extraction was performed using TRIzol (Invitrogen), according to the manufacturer’s instructions. Myobundles were entirely extracted from the hydrogels as described above and then further processed. RNA processing was performed by means of the Illustra QuickPrep Micro mRNA Purification kit (GE Healthcare) according to manufacturer’s instructions. RNA quality and quantity were determined by using an Agilent 2100 bioanalyzer (Agilent Technologies) and the Qubit Quantitation Platform (Invitrogen), respectively.

The resulting polyadenylated mRNA was further processed for library preparation using the SOLiD Whole Transcriptome Analysis Kit (LifeTechnologies), according to manufacturer’s instructions. After running on a SOLiD 5500XL DNA sequencer (Applied Biosystems), the reads were mapped to the reference human genome (hg19) using PASS [37], setting the minimal percentage identity to 90%, and allowing one gap. All samples were sequenced by the SOLiD system as described, except for AMSBIO skeletal muscle sample, which was sequenced by the Ion Proton System (ThermoFisher Scientifics). To make sure this did not represent a bias in the analysis, the Stratagene skeletal muscle sample was sequenced both with SOLiD and Ion Proton systems. Ion Proton System reads were aligned on the reference genome using a two-step procedure. At first, the reads were aligned using STAR [38]. All the reads that did not aligned during the first step were realigned with Bowtie [39] using a local alignment strategy. The counts related to each gene were computed using Htseq-count [40]. The row count matrix was normalized to take into account both the GC content biases and the different depth of coverage.

Normalization was performed using the full quantile normalization implemented in the EDASeq R package [41], while the differential expression analysis was performed with EdgeR package [42]. Genes with a p-value lower or equal 0.05 after false discovery rate correction were considered significantly differentially expressed. All figures were plotted using MATLAB R2017a. Heat maps were produced after ordering genes according to hierarchical clustering with complete linkage and Euclidean distance, each column represents the mean gene expression level of biological replicates in that condition. Skeletal muscle term in UP_TISSUE category in DAVID Bioinformatics database (v. 6.8) was used to select the subset of skeletal muscle-related genes. Statistical over-representation test was performed using PANTHER database (Released 20171205), annotation version 13.1 released on 2018-02-03.

Quantitative analysis of MHC2 (Hs00430042_m1), MHC3 (Hs01074230_m1) and dystrophin expression (Hs00758098_m1) in hES-derived 2D and 3D cultures was performed with TaqMan gene expression assay probes (Life Technologies). Reactions were performed with an ABI Prism 7000 machine and results were analyzed with the ABI Prism 7000 SDS software. GAPDH expression was used to normalize Ct values of gene expression, and data were shown as relative fold change to 2D cultured myotubes using the delta-delta Ct method.

### 2.8 Image preparation and analysis

We used ImageJ software (open-source GNU GPL v3 license) for adjustments of levels and contrast. For fluorescence intensity quantification, at least 3 fluorescence images of independent biological triplicates for each sample were converted in binary masks, then single cell fluorescence analyzed with measurement plugin of ImageJ software. Fluorescence intensity was normalized for background fluorescence. All statistical analysis were performed by using GraphPad prism 6 software. We expressed data as means ± s.e.m. We determined statistical significance by unequal variance Student’s t-test. A P value of less than 0.05 was considered statistically significant.

## 3. Results

### 3.1 3D micro-channel hydrogel design

**Fig 1:**
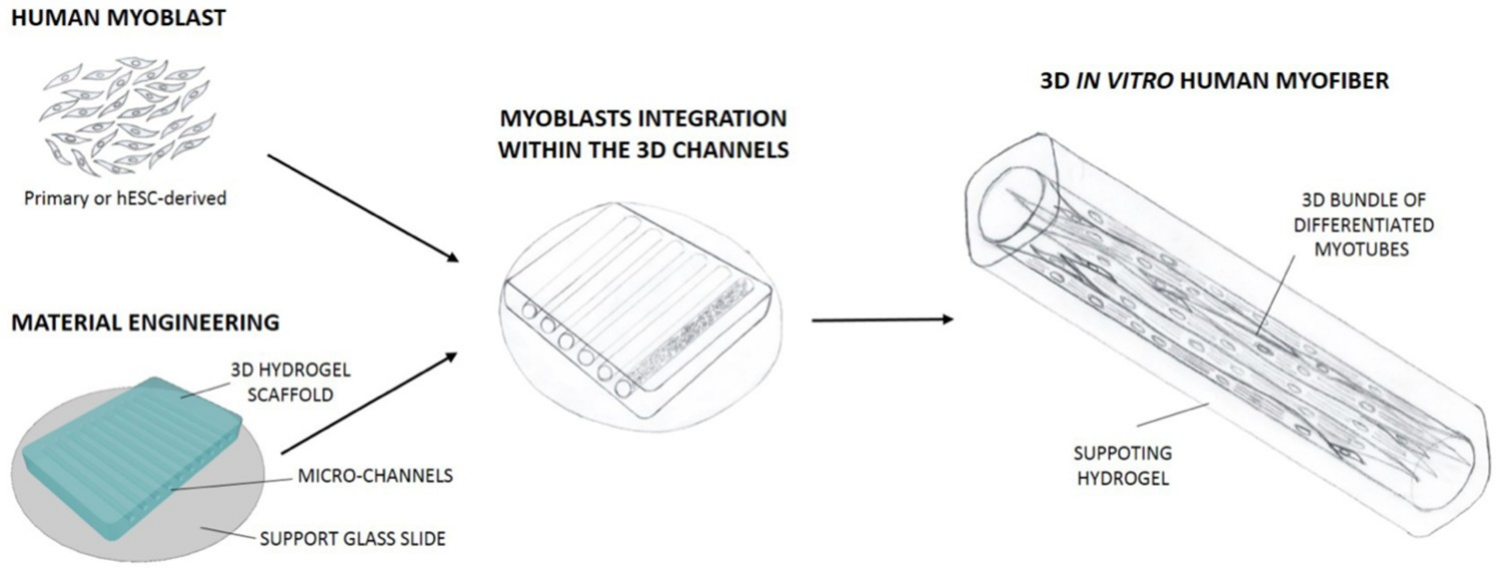
Schematic cartoon showing the cellular and engineered hydrogel strategy used to obtain human myobundles. Human primary or ES-derived myoblasts were seeded into engineered hydrogel, with optimal 3D topology and mechanical properties. Myoblasts seeded into the channels were induced to differentiate in myotubes, allowing the formation of 3D myobundle supported by the surrounding hydrogel material.

**Fig 2:**
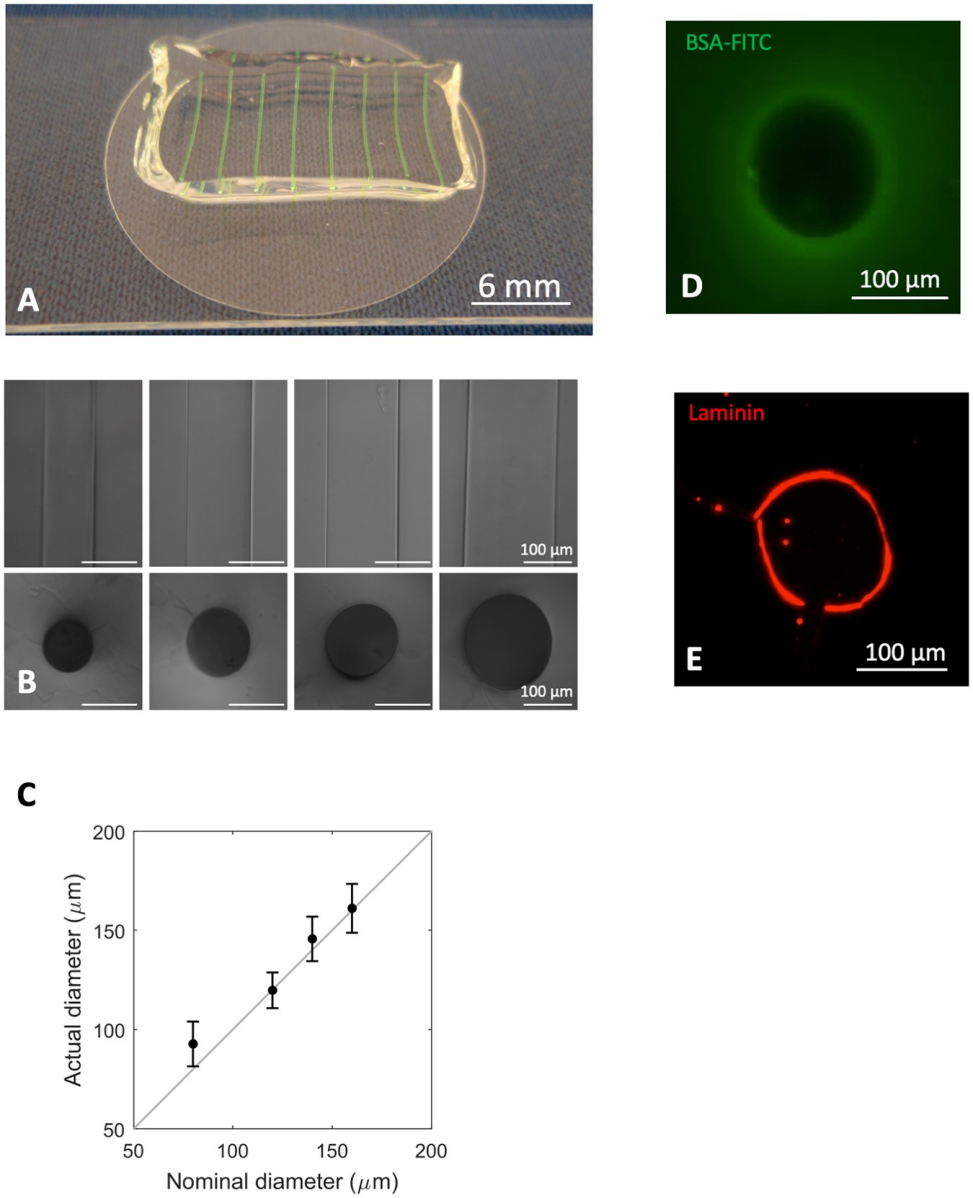
Fabrication and characterization of 3D hydrogel equipped with micrometric channels. **A.** Representative image of 3D hydrogel, in which micro-channels were loaded with a green dye. **B.** Phase contrast of micrometric channels characterized by different diameters and their transversal section. **C.** Quantification of the mean diameters of micrometric channels obtained using cylindrical fillers of different dimensions. **D.** Cross-section of a 120 μm micrometric channel loaded with a solution of BSA-FITC (green). **E.** Cross-section of a 120 μm micrometric channel coated with laminin, and revealed by immunofluorescence analysis (red).

With the aim of providing an instructive 3D culture system capable of promoting extensive myogenic cell differentiation [37], we designed a poly-acrylamide-based hydrogel with topologically organized microchannels and physio-mechanical properties resembling *in vivo* characteristics of skeletal muscle (Fig 1). In preliminary experiments, micrometric channels with diameters of 80 μm, 120 μm, 140 μm and 160 μm were generated inside the 3D hydrogels (Fig 2A–2C). The integrity of the micro-channels was demonstrated by injection and visualization of a BSA-FITC solution (Fig 2A and 2D). Both topology and mechanical properties of the substrate in which myogenic cells are cultured have been shown to strongly influence cell behaviour and myotube formation [38,39]. For this reason, we generated hydrogels that not solely mimicked the topology of a myofiber, but also displayed the optimal stiffness for human myoblast differentiation [37], which has been reported to be ≅ 15 kPa. Last but not least, besides designing our microchannels with defined biomechanics and topology, we also wanted to equip them with a proper pro-adhesive and differentiating stimulus. To this aim, their inner wall was coated with laminin (Fig 2E), as laminins are part of the basal lamina of skeletal muscle fibres and have been shown to promote myoblast adhesion and myotube formation both *in vitro* and *in vivo* [40–42].

### 3.2 Development of the 3D myogenic culture

**Fig 3:**
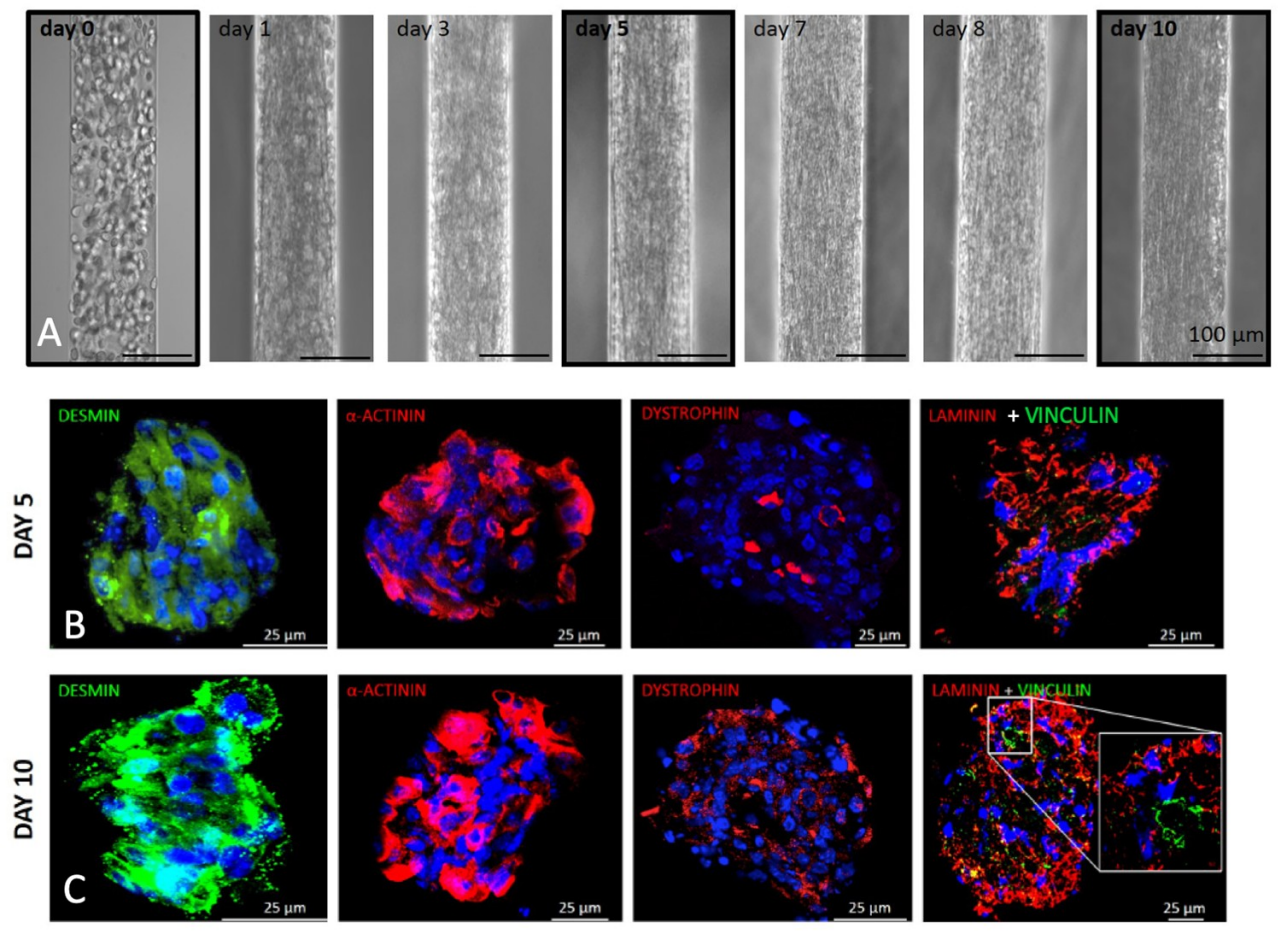
Culture of C2C12 myoblasts into 3D micrometric channels. **A.** Representative images showing the change of cell morphology during the culture of C2C12 myoblasts seeded inside the micrometric channels. **B-C.** Immunofluorescence analysis of desmin (green), α-actinin (red), dystrophin (red), laminin (red) and vinculin (green) performed on cross-cryosections derived from C2C12 3D cultures 5 (B) and 10 (C) days after myoblasts injection in the 3D micrometric channels. Nuclei were stained with Hoechst (blue).

Because of their size and their capability to differentiate into myotubes, C2C12 cells (murine myoblasts) were initially used to optimize the cell seeding conditions within the 3D micro-channels (Fig 3). The narrowest channel size did not allow a homogeneous distribution of the cell suspension upon injection, while sizes from 120 to 160 yielded comparable results. For all subsequent experiments we then chose to use the 120 μm diameter, as it was the closest to the maximum diameter of single human fibers. We devised a protocol in which by re-suspending cells at high density (2 x 10^5^ cells/μl) in 25% Matrigel we were able to homogeneously fill the channels without leakage at the ends. Moreover, with this seeding condition cells were packed densely enough to be induced to differentiate into myotubes the day after seeding (Fig 3A). In order to monitor cell behaviour during culture, time course analysis was performed on cells cultured in 3D micro-channels from the moment of seeding to the end of the experiment, after 10 days. At day 0, i.e. just after seeding, cells exhibited a round morphology and acquired an elongated morphology during the following days in culture, generating a myobundle that was in direct contact with the channel walls and became more compact starting from day 7 of culture (Fig 3A). To understand if such a 3D culture condition was able to support myogenic differentiation and myotube maturation, cross-sections of myobundles were analyzed for the expression of myogenic proteins by immunofluorescence at different time points. Myobundles displayed high levels of desmin and α-actinin already at day five, whereas at the longer time point they showed increasing levels and higher organization of three proteins typical of mature myotubes, namely dystrophin, laminin and vinculin (Fig 3B and 3C and S1 Fig).

### 3.3 Derivation of human mature myobundles using primary myoblasts

**Fig 4:**
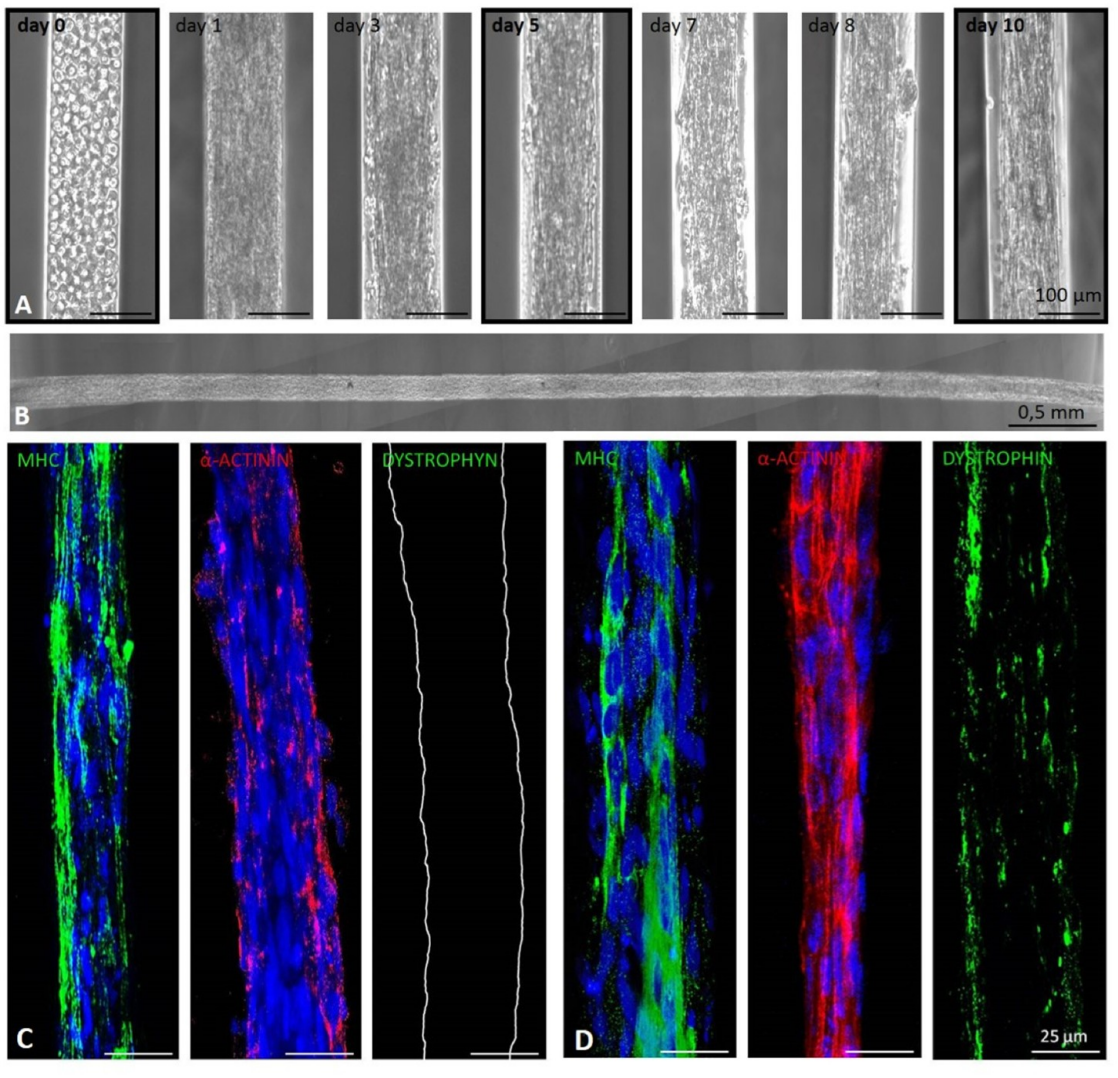
Culture of human primary myoblasts into 3D micrometric channels. **A.** Representative images showing the change of cell morphology during the culture of human primary myoblasts seeded inside the micrometric channels. **B.** Partial image of a single human myobundle upon removal from inside the hydrogel, 10 days after seeding. **C-D.** Immunofluorescence analysis performed on longitudinal sections of human myobundles using antibodies against MHC (green), α-actinin (red) and dystrophin (green) 5 (C) and 10 (D) days after myoblasts seeding in the 3D micrometric channels. Nuclei were stained with Hoechst (blue).

Based on the 3D cell culture optimization carried out with C2C12 cells, human primary myoblasts derived from biopsies of healthy donors were characterized (S2A–S2E Fig) and then seeded into 3D micro-channel hydrogels. Once again, time course analysis showed that human primary myoblasts could be successfully cultured inside the 3D micrometric channels for at least 10 days. Indeed, as seen for C2C12 cells, human myoblasts acquired an elongated morphology over time, with the formation of compact myobundles starting from day 3 of culture (Fig 4A). Importantly, our 3D culture system allowed us to obtain human myobundles of 10 mm length after 10 days of culture (Fig 4B). Moreover, spontaneous contraction of myobundles in absence of any external stimulation was observed starting from day 7 of culture (S1 Movie), when they started to detach from the micro-channel walls (Fig 4A). To assess the extent to which 3D microchannel culture was promoting myotube differentiation and maturation, human myobundles were analyzed by immunofluorescence. In agreement with the results obtained with C2C12 cells, human myobundles maturation improved over the days of culture. Expression of MHC and α-actinin was already clear after 5 days in culture (Fig 4C), but higher expression and more specific spatial distribution (including areas of striation) were observed for both proteins 10 days after seeding (Fig 4D and S2F Fig). Importantly, dystrophin was only present after 10 days of culture, reinforcing the evidence that 3D micro-channel culture allowed human myotube maturation over time (Fig 4D).

**Fig 5:**
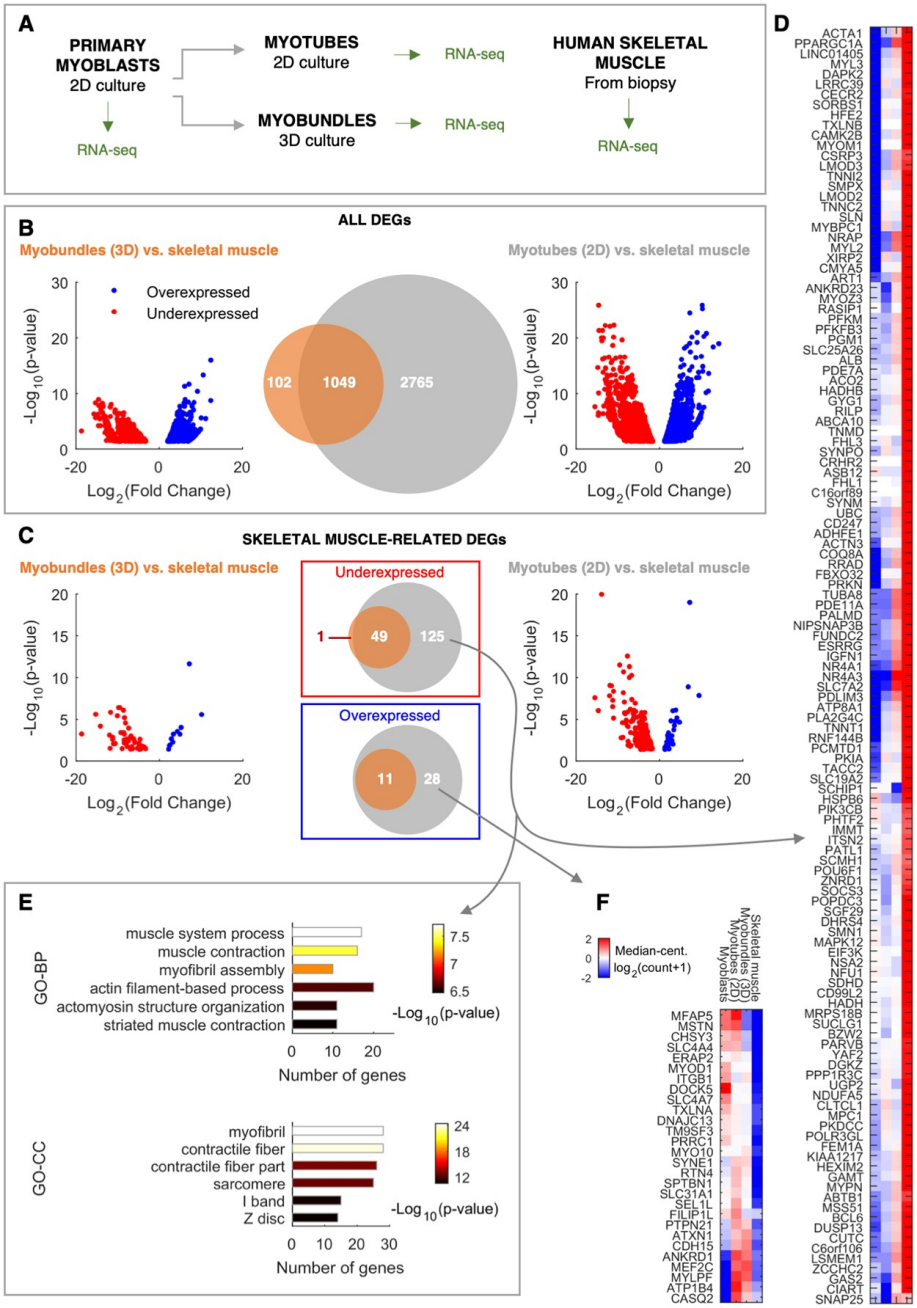
Transcriptome analysis highlights maturation of contraction machinery in 3D myobundles. **A.** Scheme of the experimental protocol to compare total mRNA profile of samples from different culture systems and stages of maturation. **B.** Comparison between all DEGs identified in the comparison between 3D myobundles and skeletal muscle, and between 2D myotubes and skeletal muscle, in terms of gene overlap (center Venn diagram) and fold change and significance (left and right Volcano plots). p-value: FDR adjusted p-value. **C.** Analysis analogous to that shown in B, but restricted to skeletal muscle-related genes. **D-E.** Heat map of gene expression and functional annotation of muscle genes underexpressed in 2D myotubes respect to skeletal muscle, respectively. GO-BP: Gene Ontology—Biological Process. GO-CC: Gene Ontology—Cellular Component. **F.** Heat map of gene expression of muscle genes overexpressed in 2D myotubes respect to skeletal muscle. Color bar and sample name order refer to both D and F heat maps.

To further investigate the role of our 3D culture system in promoting the functional maturation of human myobundles, a transcriptomic analysis was performed comparing myoblasts, myotubes cultured in conventional 2D systems (2D myotubes), myobundles cultured in our 3D microchannels and adult human skeletal muscle (Fig 5A). Firstly, a differential expression analysis of cultured cells was performed with respect to adult skeletal muscle. Interestingly, the number of significant differentially expressed genes (DEGs) identified between 2D myotubes and skeletal muscle was three times higher than that found between myobundles and skeletal muscle (Fig 5B), with as few as 9% of total DEGs observed only in the comparison between myobundles and skeletal muscle (Fig 5B). These results were further corroborated by Volcano plots, which showed higher significance (lower adjusted p-value) of DEGs identified between 2D myotubes and skeletal muscle (Fig 5B).

Because skeletal muscle samples can contain other cell type impurities due to the experimental procedure of isolation, we then focused our analyses on skeletal muscle-related genes, which can better capture aspects of myotube maturation we are interested in. In both culture systems (2D and 3D), most of DEGs were underexpressed, rather than overexpressed, respect to the levels observed in skeletal muscle (Fig 5C). Moreover, 3D myobundles differed from skeletal muscle only for a subset of the DEGs identified between 2D myotubes and skeletal muscle (Fig 5C). This was better visualized in the heat maps shown in Fig 5D and 5F, where DEGs under- or over-expressed exclusively in the 2D culture system respect to skeletal muscle are plotted. These DEGs are instead similar between 3D myobundles and skeletal muscle. Interestingly, the list of muscle-related DEGs underexpressed only in the 2D system is enriched in genes functionally and structurally related to muscle contraction, pointing out to a higher level of functional maturation in the 3D system (Fig 5E).

A direct comparison between the expression profiles of 2D myotubes and 3D myobundles by hierarchical clustering analysis shows the distance among the different samples analyzed and confirms our conclusion that 3D myobundles have an expression profile more similar to that of skeletal muscle, compared to 2D myotubes which are closer to myoblast condition (S3 Fig). This was observed both considering all DEGs between 3D myobundles and 2D myotubes (S3A Fig), and also restricting the analysis to muscle-related DEGs (S3B Fig). Interestingly, DEGs over-expressed in 3D myobundles respect to 2D myotubes are over represented in some categories already known to be involved in skeletal muscle maturation, such as those related to cholesterol metabolism and extracellular matrix organization (S1 Data).

### 3.4 Human 3D myobundles can be derived by using hESC-derived myoblasts

**Fig 6:**
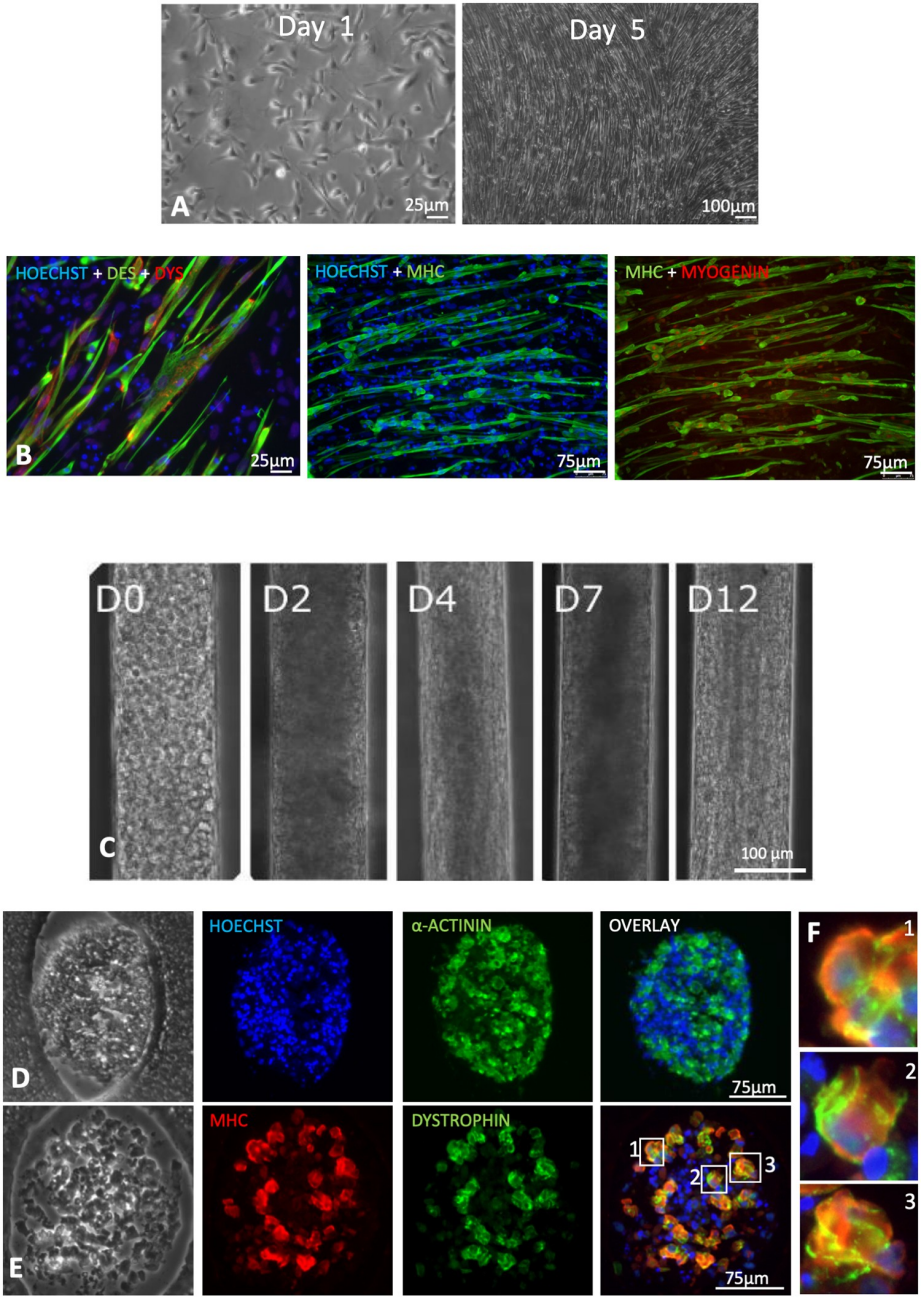
Characterization of hESC myoblasts and their culture inside 3D micrometric channels. **A.** Phase contrast representative images of primary myogenic cells cultured under proliferating condition at 1 and 5 days after seeding. **B.** Representative images of immunofluorescence analysis for desmin (green) and dystrophin (red), for MHC (red) and for MHC and myogenin. Nuclei were stained with DAPI (blue). **C.** Time course of hESC myoblasts culture inside the micrometric channels. **D-E.** Immunofluorescence analyses of MHC, α-actinin and dystrophin on 3D myotubes bundles obtained 12 days after myoblasts injection in the micrometric channels. **F.** Magnification of myotubes showing the co-expression of MHC and Dystrophin, this latter localized preferentially at the membrane.

Our last set of experiments aimed at verifying if the 3D culture system could be successfully used with human myoblasts derived from human embryonic stem cells (hESCs). In particular, we wanted to determine if we could trigger the differentiation of hESC-derived myoblasts towards myobundles and if their maturation level was comparable to that obtained with primary cells. hESC-derived myoblasts were firstly characterized under standard 2D culture condition in proliferation or differentiation conditions (Fig 6A and 6B), then seeded into the channels as described above and analysed at different time points. Once inside the channels, hESC-myoblasts displayed a morphological progression similar to that observed for C2C12 cells and human primary myoblasts (Fig 6C). Evidence of myotube maturation was observed over the period of culture, as shown by the identification of MHC, α-actinin and dystrophin in myobundles (Fig 6C–6E). Importantly, dystrophin appeared to be correctly localized at the membrane (Fig 6F). When compared to conventional 2D cultured myotubes, quantitative RNA expression analysis showed that 3D cultured cells display increased expression of MHC2, MHC3 and dystrophin (S4A Fig). In accordance with these observations, quantification of intensity fluorescence confirmed increased dystrophin levels in hESC-myoblasts cultured in 3D, when compared to 2D conventional cultures (S4B Fig). Moreover, a significant increase of the fusion index was observed when cells were cultured in 3D culture conditions, compared to 2D cultures (S4C Fig). However, despite the longer culture period of hiPSC-derived myoblasts compared to primary myoblasts, no spontaneous contraction could be observed in 3D myobundles derived from hESC-Mbs.

## 4. Discussion

In recent years the importance of generating 3D culture system for better mimicking skeletal muscle myogenesis and physiology *in vitro* has been well demonstrated. The most common approaches that allow the creation of skeletal muscle constructs are based on the use of 3D culture conditions in which myogenic cells self-assemble in a bulk biomaterial. Here we successfully generated human myobundles culturing human myogenic cells in poly-acrylammide-based hydrogel with controlled, topologically organized microchannels and physiological mechanical properties resembling *in vivo* characteristics of skeletal muscle. Poly-acrylammide-based hydrogel represents a well-defined system that can provide accurate modulation of mechanical properties, being completely cell repellent and thus allowing the generation of multiple separated myobundles in the same 3D culture system. Amongst the different micrometric channel diameters that could be generated and coated with laminin, proper cell distribution was achieved with channels with a diameter ≥120μm; as we wanted to stay as close as possible to the size of single muscle fibers, we decided to use the 120μm lumen for all subsequent experiments. Geometrical confinement was able to generate packed and aligned 3D myobundles whose myogenic differentiation increased with culture time, both with murine (C2C12) or human primary myoblasts. Our results are in agreement with other studies in which closely packed C2C12 cells yielded better aligned myotubes as the level of 3D confinement was increased–i.e. reducing the diameter [38]. Moreover, our 3D culture system also sustains myotube maturation independently from the seeded cell types, as demonstrated by the expression and correct localization of proteins produced upon myotube maturation, such as dystrophin, laminin and vinculin. Altogether these data indicated that the 3D hydrogels characterized by optimal stiffness for human skeletal muscle differentiation and equipped with laminin-absorbed channels were able to sustain human myoblast differentiation and maturation, leading to the generation of aligned myotubes organized in stable 3D bundles that can reach ~10 mm of length. Such results also open the future possibility to adapt our 3D culture approach for generating myobundles of longer lengths (> 10mm), thus eventually opening novel perspective for translational approaches.

Despite the above similarities, differences were observed among the different cell sources used. In particular, myobundles derived from human primary cells showed higher maturation compared to C2C12 cells cultured in the same condition; also, spontaneous contraction was observed only in the former myobundles. Interestingly, human primary myoblasts-derived myobundles started to detach from the micro-channel walls when spontaneous contraction was observed. However, such detachment–which was likely due to the mechanical stress itself–did not prevent myobundle maintenance until the last experimental time point–i.e. 10 days. This effect could be likely due to the elastic modulus and compliance of the surrounding hydrogel which can support the integrity of the myobundle. As shown in the accompanying movie, spontaneous contraction was not synchronous along the whole construct bas was instead seen in specific zones. Such observation was not unexpected, though, as myotubes do not form a functional syncytium (i.e., there are no gap junctions) and hence in the absence of external stimuli there is no way to spread the calcium spikes amongst neighbouring areas. The ability of myobundles to undergo maturation was further confirmed by transcriptome analysis. Indeed, a higher similarity of gene expression between myobundles obtained in our 3D culture system and skeletal muscle was observed, in respect to myotubes derived in conventional 2D culture systems and skeletal muscle. Looking at genes specifically related to skeletal muscle, the majority of the DEGs identified were underexpressed in cultured cells when compared to skeletal muscle. This is in accordance with the incomplete maturation of the *in vitro* system, when compared to *ex-vivo* tissue. However, essentially all the DEGs identified between 3D myobundles and skeletal muscle were a subset of those revealed between 2D myotubes and skeletal muscle. These data strongly suggests that our 3D culture supports better myotube maturation in comparison to conventional 2D myotube culture. In particular, at the transcriptomic level, cells cultured in the 2D culture condition showed reduced expression of genes involved in structural and functional maturation of the contraction machinery, as well as in 3D organization (extracellular matrix organization) and skeletal muscle function, when compared to the 3D myobundle. Altogether, this analysis supports the idea that our 3D culture system allows human myotube maturation.

Human primary myoblasts ideally represent the best cell type to be used for skeletal muscle modeling and engineering. However, the scarce availability of human skeletal muscle biopsies together with the limitation imposed by *in vitro* expansion of primary myoblasts render this cell type poorly suitable for the development of *in vitro* models for high throughput drug screening, or the development of cell-based therapies. In this regard, the most promising cell source is represented by human pluripotent stem cells (hPSCs), which not only have an unlimited proliferative potential but also can be induced to differentiate into myogenic cells while maintaining pathological phenotypes. Besides, hPSC can also be subjected to genome editing to study disease variants in the same genetic background or correct underlying mutations [34]. Both induced pluripotent stem cell-derived and hESC-derived myoblasts cultured in a 3D bulk hydrogel were shown to be able to self-assemble and differentiate into mature and functional myotubes [34,35]. Based on this, we investigated whether our 3D in vitro culture could also sustain myogenic differentiation and myobundle formation of hESC-derived myoblasts. Interestingly, and in agreement with data obtained with C2C12 and primary myoblasts, compact MHC^+^ bundles of myotubes showing correct deposition of dystrophin were obtained by culturing hESC-derived myoblasts into the microchannels of our 3D hydrogels. Importantly, immunofluorescence and gene expression analyses strongly suggested that 3D cultured hESC-derived myoblasts were indeed able to proceed toward myotube maturation. However, a slightly longer period of culture was necessary to obtain compact human myobundles from hESC-derived myoblasts, specifically, 12 rather than 10 days; besides, myobundles from hESC-derived myoblasts did not show spontaneous contraction. Such discrepancy observed between human primary myoblasts-derived and hESC-myobundles could be due to the intrinsic difference existing between these two cell types. Indeed, it is known that hPSC-derived myotubes as well as myobundles have been shown to be more developmentally immature than primary-derived myotubes [34,43–45].

## 5. Conclusion

Here we have demonstrated that individual human myobundles characterized by ~10 mm of length with a high 3D organization and degree of maturation can be derived *in vitro* from both human primary myoblasts and hESC-derived myoblasts, by coupling topographic and mechanical properties of a 3D cell culture system. Such *in vitro* culture strategy represents a powerful tool for modelling human skeletal muscle and could represent a novel approach for investigating healthy and diseases skeletal muscle. Thanks to the hydrogel mechanical support, the developed strategy allows the generation of contracting myobundles that do not require any anchor at their extremities. This in turn would allow the handling of single bundles whose size fall in the range of mammalian muscle fibers and are free from the need of external supports. Moreover, addition of others cellular or tissue-specific components, e.g., tendon structures at the extremities, as well as mechanical and/or electrical stimuli, should be relatively straightforward and could lead to even better models of engineered human skeletal muscle *in vitro*.

## Supporting information

S1 Fig Quantification of fluorescence intensity for actinin and dystrophin in C2C12 cells cultured in 3D 5 or 10 days after seeding. Data are shown as mean ± s.d. of 3 independent replicates; Student’s t-test was used; *P< 0.05; **P< 0.02.
(PDF)

S2 Fig Characterization of primary human myoblasts. **A-D.** Phase contrast representative images of primary myogenic cells cultured under proliferating condition at 1 (A) and 2 (B) days after seeding, at confluence (C) and after 7 days from from switching to differentiation medium in standard 2D culture dishes (D). **E.** Representative images of immunofluorescence analysis for desmin (green), ⍺-actinin (red) and myosin heavy chain (MHC, red). Nuclei were stained with hoechst (blue). **F.** Quantification of fluorescence intensity for actinin and dystrophin in primary human myoblasts cultured as 3D myobundles 5 or 10 days after seeding. Data are shown as mean ± s.e.m. of 3 independent replicates; *P< 0.05; **P< 0.02 with Student’s t-test.
(PDF)

S3 Fig Hierarchical clustering analysis. (A) Heat map and clustergrams of all DEGs identified between myobundles (3D) and myotubes (2D) samples. (B) Same as (A), restricting the analysis to muscle-related DEGs.
(PDF)

S4 Fig Quantitative comparison between hES-derived 2D myotubes and 3D myobundles. **A.** qPCR analysis of myosin heavy chain 3 (MYHC3), myosin heavy chain 2 (MYHC2), and dystrophin (DMD) expression between 2D myotubes (dotted line) and 3D myobundles obtained from hES-derived myoblasts, 12 days after switching to differentiation medium. Expression levels were normalized against GAPDH. **B.** Quantification of fluorescence intensity for dystrophin between 2D or 3D cultures of hPSC-derived myoblasts 12 days after seeding. Data are shown as mean ± s.e.m. of 3 independent replicates; *P< 0.05, with Student’s t-test. **C.** Comparison of fusion index (i.e., number of nuclei within myotubes over the total number of nuclei) between the same two conditions described in B. Data are shown as mean ± s.e.m. of 3 independent replicates; **P< 0.02, with Student’s t-test.
(PDF)

S1 Movie (AVI)

S1 Data (XLSX)
