# Supplementary material for: Engineering a 3D in vitro model of human skeletal muscle at the single fiber scale
Source: PLoS One. 2020 May 6;15(5):e0232081. doi: 10.1371/journal.pone.0232081 (PMC7202609; doi:10.1371/journal.pone.0232081)
Supplement: S1 Fig — Data are shown as mean ± s.d. of 3 independent replicates; Student’s t-test was used; *P< 0.05; **P< 0.02. (PDF) [file pone.0232081.s001.pdf]

Supplementary Figure S1

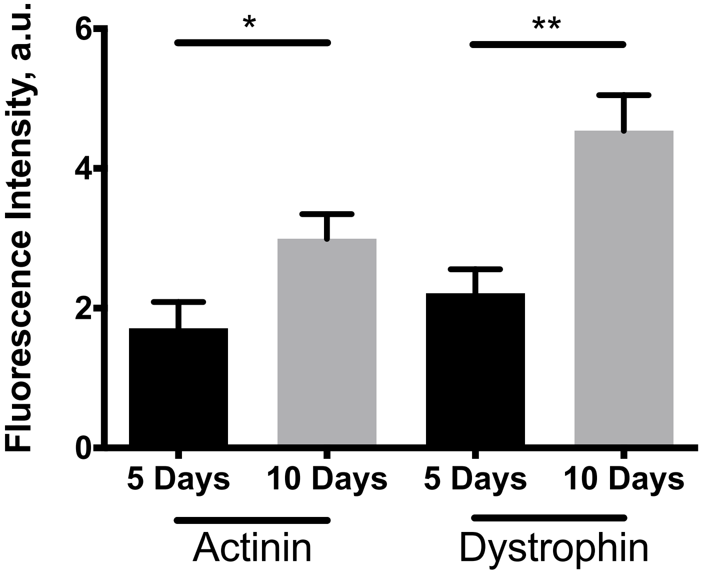

Supplementary Figure S1. Quantification of fluorescence intensity for actinin and dystrophin in C2C12 cells cultured in 3D for 5 or 10 days after seeding. Data are shown as mean ± s.e.m. of 3 independent replicates; **P*< 0.05; ***P*< 0.02 with Student's t-test.
