# Supplementary material for: Engineering a 3D in vitro model of human skeletal muscle at the single fiber scale
Source: PLoS One. 2020 May 6;15(5):e0232081. doi: 10.1371/journal.pone.0232081 (PMC7202609; doi:10.1371/journal.pone.0232081)
Supplement: S2 Fig — A-D. Phase contrast representative images of primary myogenic cells cultured under proliferating condition at 1 (A) and 2 (B) days after seeding, at confluence (C) and after 7 days from from switching to differentiation medium in standard 2D culture dishes (D). E. Representative images of immunofluorescence analysis for desmin (green), ⍺-actinin (red) and myosin heavy chain (MHC, red). Nuclei were stained with hoechst (blue). F. Quantification of fluorescence intensity for actinin and dystrophin in primary human myoblasts cultured as 3D myobundles 5 or 10 days after seeding. Data are shown as mean ± s.e.m. of 3 independent replicates; *P< 0.05; **P< 0.02 with Student’s t-test. (PDF) [file pone.0232081.s002.pdf]

Supplementary Figure S2

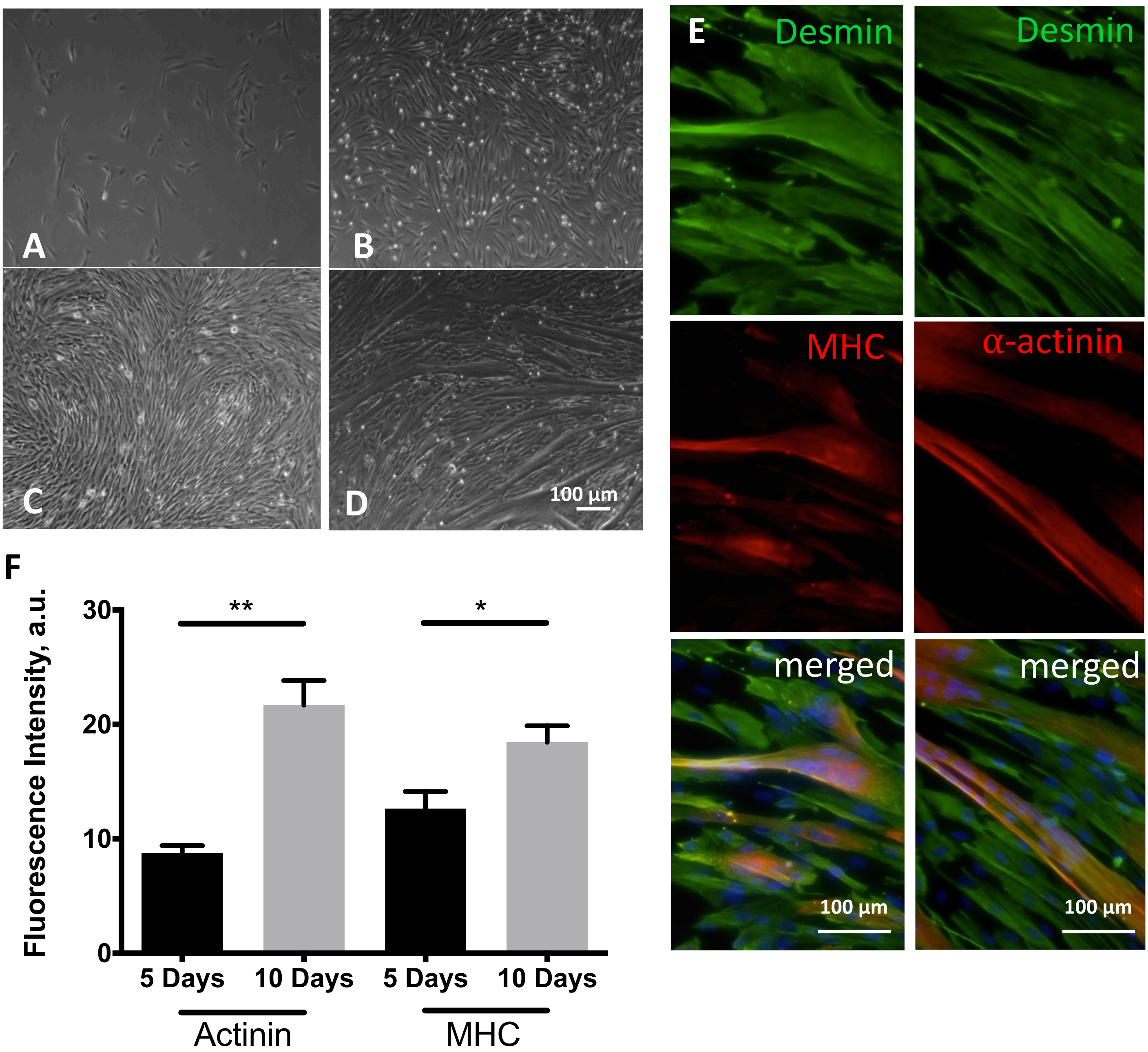

Supplementary Figure S2: Characterization of primary human myoblasts. A-D. Phase contrast representative images of primary myogenic cells cultured under proliferating condition at 1 (A) and 2 (B) days after seeding, at confluence (C) and after 7 days from from switching to differentiation medium in standard 2D culture dishes (D). **E.** Representative images of immunofluorescence analysis for desmin (green), α-actinin (red) and myosin heavy chain (MHC, red). Nuclei were stained with hoechst (blue). **F.** Quantification of fluorescence intensity for actinin and dystrophin in primary human myoblasts cultured as 3D myobundles 5 or 10 days after seeding. Data are shown as mean ± s.e.m. of 3 independent replicates; $*P< 0.05$; $**P< 0.02$ with Student's t-test.
