# Supplementary material for: Engineering a 3D in vitro model of human skeletal muscle at the single fiber scale
Source: PLoS One. 2020 May 6;15(5):e0232081. doi: 10.1371/journal.pone.0232081 (PMC7202609; doi:10.1371/journal.pone.0232081)
Supplement: S3 Fig — (A) Heat map and clustergrams of all DEGs identified between myobundles (3D) and myotubes (2D) samples. (B) Same as (A), restricting the analysis to muscle-related DEGs. (PDF) [file pone.0232081.s003.pdf]

## Supplementary Figure S3

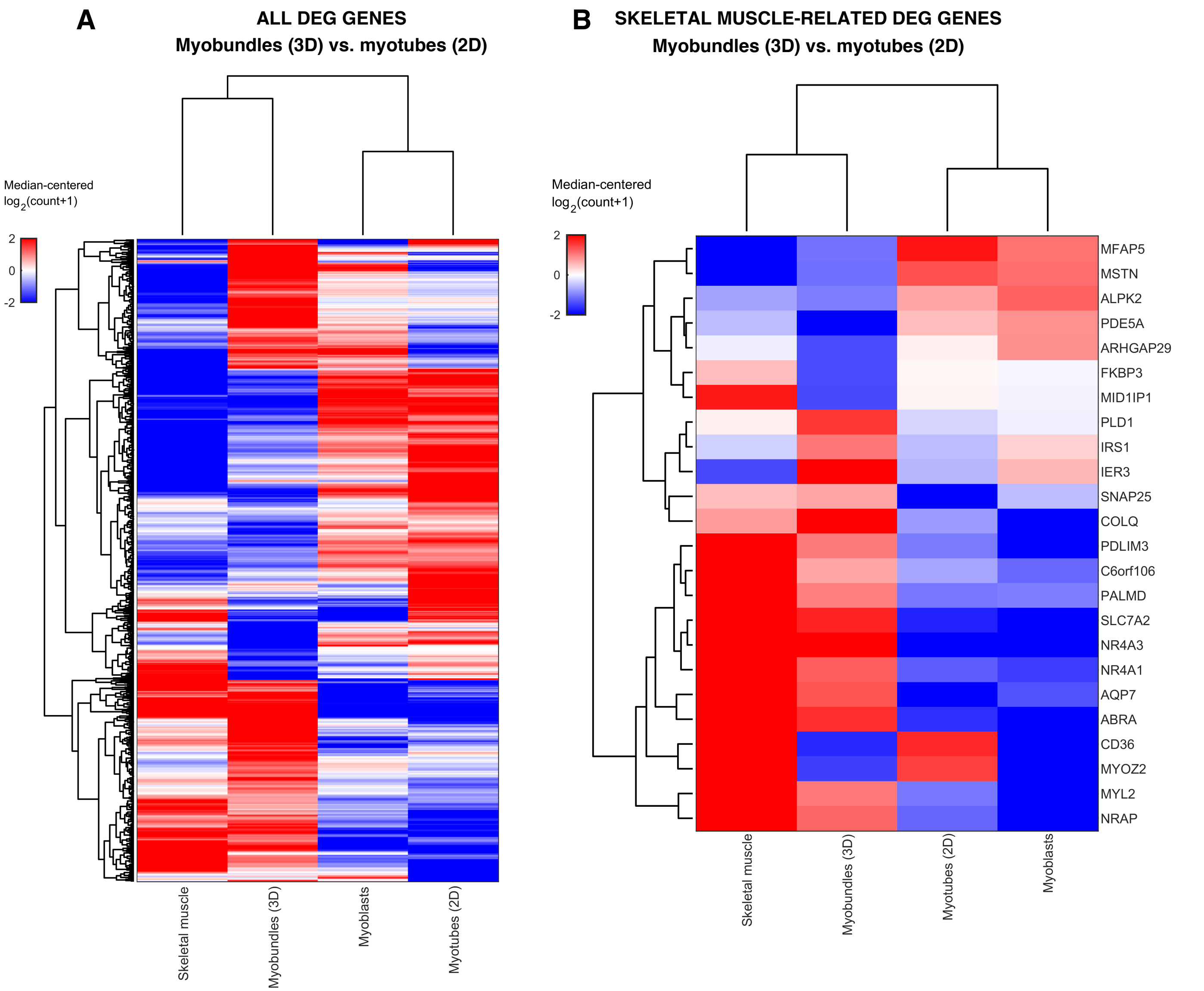


**Supplementary Figure S3: Hierarchical clustering analysis. A.** Heat map and clustergrams of all DEGs identified between myobundles (3D) and myotubes (2D) samples. **B.** Same as (A), restricting the analysis to muscle-related DEGs.
