# Supplementary material for: Engineering a 3D in vitro model of human skeletal muscle at the single fiber scale
Source: PLoS One. 2020 May 6;15(5):e0232081. doi: 10.1371/journal.pone.0232081 (PMC7202609; doi:10.1371/journal.pone.0232081)
Supplement: S4 Fig — A. qPCR analysis of myosin heavy chain 3 (MYHC3), myosin heavy chain 2 (MYHC2), and dystrophin (DMD) expression between 2D myotubes (dotted line) and 3D myobundles obtained from hES-derived myoblasts, 12 days after switching to differentiation medium. Expression levels were normalized against GAPDH. B. Quantification of fluorescence intensity for dystrophin between 2D or 3D cultures of hPSC-derived myoblasts 12 days after seeding. Data are shown as mean ± s.e.m. of 3 independent replicates; *P< 0.05, with Student’s t-test. C. Comparison of fusion index (i.e., number of nuclei within myotubes over the total number of nuclei) between the same two conditions described in B. Data are shown as mean ± s.e.m. of 3 independent replicates; **P< 0.02, with Student’s t-test. (PDF) [file pone.0232081.s004.pdf]

**Supplementary Figure S4**

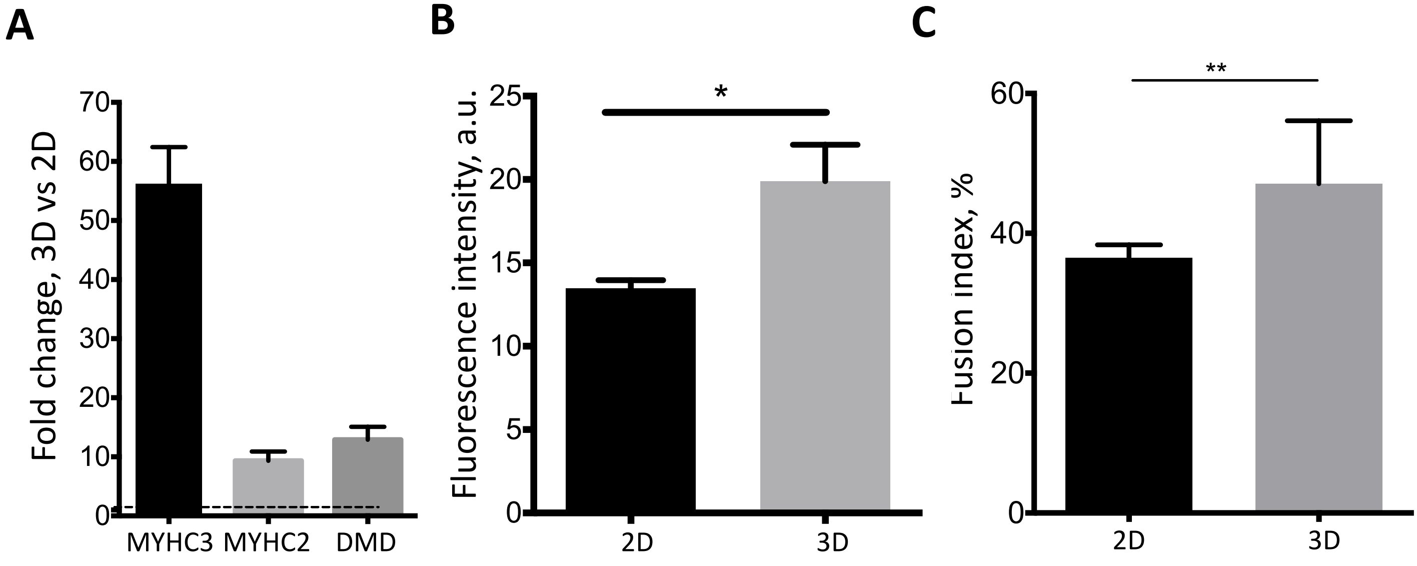


**Supplementary Figure S4. Quantitative comparison between hES-derived 2D myotubes and 3D myobundles. A.** qPCR analysis of myosin heavy chain 3 (MYHC3), myosin heavy chain 2 (MYHC2), and dystrophin (DMD) expression between 2D myotubes (dotted line) and 3D myobundles obtained from hES-derived myoblasts, 12 days after switching to differentiation medium. Expression levels were normalized against GAPDH. **B.** Quantification of fluorescence intensity for dystrophin between 2D or 3D cultures of hPSC-derived myoblasts 12 days after seeding. Data are shown as mean ± s.e.m. of 3 independent replicates; *$P< 0.05$, with Student's t-test; **C.** Comparison of fusion index (i.e., number of nuclei within myotubes over the total number of nuclei) between the same two conditions described in B. Data are shown as mean ± s.e.m. of 3 independent replicates; **$P< 0.02$, with Student's t-test.
